# Management of Patients With Normal Physical Exams and Ultrasound Evidence of Isolated Septal and Punctate Penile Scarring

**DOI:** 10.1016/j.esxm.2021.100346

**Published:** 2021-05-30

**Authors:** Noah Stern, Nahid Punjani, Gerald Brock

**Affiliations:** 1 Division of Urology, London Health Sciences Centre , London, Ontario, Canada; 2 Division of Urology, Weill Cornell Medicine , New York, NY, USA; 3 Division of Urology, Western University Ringgold Standard Institution, London, Ontario, Canada

**Keywords:** Erectile Dysfunction, Ultrasonography, Doppler, Peyronie's Disease

## Abstract

**Introduction:**

Atypical penile tunical lesions including isolated septal (ISS) and punctate scarring (PS) are an under recognized and difficult to treat subset of Peyronie's disease (PD) that often present with normal physical exams. Current guidelines provide little direction in the treatment of these men.

**Aim:**

To review the results of our treatment approach in men with ISS and PS.

**Methods:**

Data from all men undergoing duplex ultrasonography for either Peyronie's disease and/or erectile dysfunction over a 3-year period were reviewed. All men with ISS or PS and normal physical exams were included. First- and second-line treatment preferences and satisfaction with treatment in men with ISS and PS were retrospectively reviewed. Logistic regression was used to investigate associations between scar features and treatment preference

**Main Outcome Measures:**

Treatment preference patterns, treatment satisfaction.

**Results:**

A total of 217 men with ISS and 197 men with PS were identified. Of these, 71 ISS and 86 PS patients had normal physical exams. Majority of men in both ISS (70.4%) and PS (81.4%) cohorts initially opted for non-invasive management through either observation, oral therapy, or traction therapy. After initial management 84.5% of ISS and 93% of PS patients were satisfied with their results. A significant trend toward inflatable prostheses as second line therapy was seen in men with PS.

**Conclusions:**

There is a mounting need for clinical guidance in order to best manage men with atypical PD in the absence of societal guidelines and high-quality studies. This series provides guidance to clinicians on the management of these men, suggesting that conservative therapy and education may be sufficient. A standardized approach of increasing invasiveness showed reasonable rates of satisfaction with minimally invasive therapies playing a prominent role. **Stern N, Punjani N, Brock G, Management of Patients With Normal Physical Exams and Ultrasound Evidence of Isolated Septal and Punctate Penile Scarring. Sex Med 2021;9:100346**

## INTRODUCTION

Peyronie's disease (PD) is a benign disorder resulting in penile deformity caused by alterations of the fibro-elastic tunica albuginea.^1^ While traditionally diagnosed by history and physical exam demonstrating penile curvature and a palpable plaque, there is growing evidence that a subset of patients presenting with PD-like symptoms without the pathognomonic exam findings.^2^, ^3^, ^4^ Diagnosis of these patients may require ultrasonography to identify their isolated septal scars – penile scars involving only the penile septum (ISS) or punctate scars – small calcifications (<3mm) throughout the corpora without an obvious plaque (PS). Current treatment algorithms proposed by the American and European urological societies often require clinicians to identify and target a palpable lesion for treatment leaving little guidance for the management of these patients.^1^^,^^5^

Our center recently published the largest series of patients with atypical and non-palpable plaques.^2^ This report summarizes our treatment approach and results on men who present with normal physical exams, but evidence of ISS or PS on ultrasonography. Our aim is to give guidance to clinicians treating this unusual and poorly understood population, in the absence of any prospective or higher quality recommendations. Our intention is to investigate the role of penile ultrasonography in men with ED and normal physical exams and a structured treatment algorithm of progressively increasing invasiveness in men with PS and ISS

## MATERIALS AND METHODS

**Figure 1:**
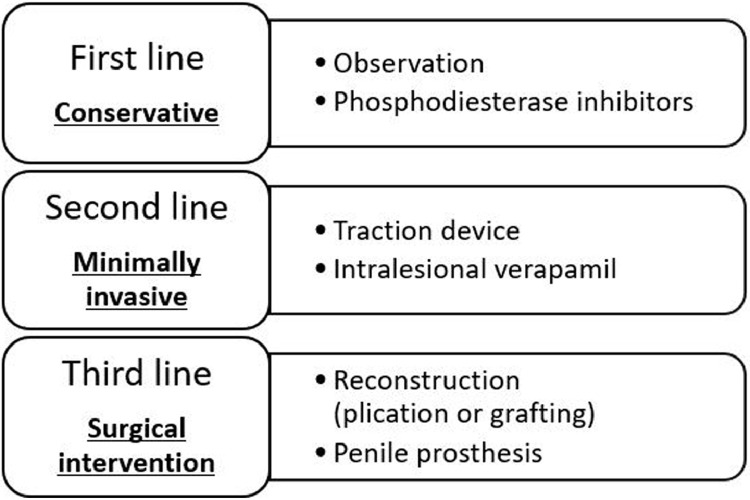
Treatment algorithm for men presenting with septal (ISS) or punctate (PS) scarring and a normal physical exam

Detailed methodology was previously described in our published work.^2^ In summary, this study involves all men referred to a single urologist at a tertiary care center with complaints of PD or erectile dysfunction (ED) who underwent duplex ultrasonography over a 3-year period with a normal physical exam and evidence of either ISS or PS. Patient demographics, examination, ultrasound findings, and treatments were recorded. Ultrasonography was performed using the linear 12.5Hz linear probe of a Phillips iU22 ultrasound machine (Philips Bothell, USA) with and without an artificial erection stimulated by intracavernosal injection of prostaglandin E-1. Both physical exam and ultrasonography was performed by the surgeon. A stepwise approach of increasingly invasive options was offered at our facility, starting with conservative options including observation and medical management, followed by minimally invasive treatments including traction devices and intralesional or intracorporeal injections, finally surgical interventions such as reconstruction or prostheses were offered (Figure 1). As erectile dysfunction is largely a quality of life concern we defined satisfaction as when men deferred further treatments options indicating they were either satisfied with their sexual function or the invasiveness of the treatments exceeded the impact of the disease on their quality of life.

Multivariable logistic regression models were used to identify treatments which were associated with either punctate or septal scarring as compared to all men with normal physical exams. Stata 14.1 (StataCorp, Texas USA) was used to perform all analyses. The study was approved by the University Research Ethics Board.

## RESULTS

A retrospective analysis identified 722 men with first time duplex ultrasounds and complete clinical data over a three-year period. Of these men, 217 (30.1%) had ISS and 197 (27.3%) had PS. Normal physical exams were seen in 71 (33%) of men with ISS and 86 (44%) of men with PS. Patient demographics, presentation and ultrasound findings, and treatment modalities and trends are shown in Tables 1 and 2.

**Table 1:** Patient demographics, presentation, ultrasound findings, and treatment of men presenting with isolated septal or punctate scarring and a normal physical exam

	Isolated septal scarring		Punctate scarring	
	n = 71		n = 86	
	n	%	n	%
Demographics				
Median age	50	IQR (43-58)	52	IQR (40-61)
Median BMI	28	IQR (25-30)	28	IQR (24-31)
Evaluation of Peyronie's disease	44		40	
Evaluation for erectile dysfunction	47		65	
Curvature				
None	44		65	
Mild	8		8	
Moderate	10		6	
Severe	9		7	
Plaque measurements				
Mean thickness (mm)	3.3	stdev: (2.3)	1.1	stdev: 0.5
Mean width (mm)	3.5	stdev: (2.8)	1.3	stdev: 1.4
Treatment				
*Primary treatment*				
Observation	20	28.2%	49	57.0%
Phosphodiesterase inhibitors	29	40.8%	18	20.9%
Traction	1	1.4%	3	3.5%
Intralesional verapamil	10	14.1%	4	4.7%
Reconstruction	8	11.3%	8	9.3%
Inflatable penile prosthesis	3	4.2%	4	4.7%
*Secondary treatment*				
Observation	60	84.5%	80	93.0%
Phosphodiesterase inhibitors	6	8.5%	1	1.2%
Traction	0	0.0%	0	0.0%
Verapamil	3	4.2%	1	1.2%
Reconstruction	1	1.4%	0	0.0%
Inflatable penile prosthesis	1	1.4%	4	4.7%

BMI = body mass index; IQR = interquartile range; Stdev = standard deviation.

**Table 2:** Impact of punctate scarring and septal scarring on choice of first line and second line treatment options

	Septal scarring		Punctate scarring	
	OR	95% CI	OR	95% CI
First Line				
Intralesional verapamil	2.53*	0.94	0.55-1.61	1.48-4.33
PDE5 inhibitors	2.32*	1.1	0.67-1.80	1.40-3.85
Traction device	0.88	2.39	0.67-8.54	0.23-3.30
Reconstruction	1.93*	1.22	0.64-2.35	1.00-3.73
IPP	1.43	0.73	0.29-1.84	0.59-3.46
Observation	0.89	0.84	0.51-1.39	0.52-1.52
Second line				
Intralesional verapamil	2.57	0.57	0.16-2.04	0.93-6.80
PDE5 inhibitors	1.81	1.17	0.51-2.67	0.84-3.90
Traction device	1.53	1.71	0.65-4.49	0.59-3.97
Reconstruction	2.96*	1.31	0.54-3.14	1.32-6.66
IPP	1.04	3.42*	1.43-8.20	0.39-2.77
Observation	1.24	1.19	0.81-1.73	0.86-1.81

CI = confidence interval; OR = odds ratio.

⁎ *P* < 0.01.

### Isolated septal scarring

Most patients with ISS preferentially chose non-invasive therapies with either observation (57.0%) or oral therapy (20.9%). Most men (93.0%) were satisfied after initial therapy and opted for no further treatment while a small subset (4.7%) opted for surgical intervention. For primary treatment, a significant preference for intralesional verapamil (OR 2.53 CI 1.48-4.33 p<0.01), oral therapy (OR 2.32 CI 1.40-3.85, p<0.01), and for surgery with penile plication as both a first line (OR 1.93 CI 1.0-3.73, p<0.01) and second line (OR 2.96 CI 1.32-6.66, p<0.01) therapy was observed.

### Punctate scarring

Similar to men with ISS, most patients with PS opted for conservative first line therapy of either observation (28.2%) or oral therapy (40.8%), while 11 (15.5%) proceeded directly with surgical management. Following primary therapy, most men (84.5%) sought no further treatment. Only 9 (12.7%) sought further therapy with 2 (2.8%) opting for surgical intervention. A significant trend toward inflatable prostheses as second line therapy was seen in this population (OR 3.42 CI 1.43-8.2, P<0.05).

## Discussion

Peyronie's disease poses a significant impact to the quality of life of both patients and their partners.^1^^,^^5^ Diagnosis is historically based on history and physical exam. While these modalities may be adequate for the traditional patients with palpable plaques, a subset will have normal exams and atypical scarring that can be difficult or impossible to detect in the absence of advanced imaging. Duplex ultrasonography has permitted us to characterize and offer treatment strategies for this subset of patients in the absence of higher quality recommendations. Our approach of increasingly invasive management options showed reasonable levels of satisfaction in both these populations with 84% of patients with ISS and 93% of patients with PS opting for no further treatment after first line therapy.

Standard use of duplex ultrasound remains controversial in the management of Peyronie's disease, as it is often insensitive and imprecise in the detection and characterization of plaques. Current European guidelines recommend against its use while the American Urological Association acknowledges it as an option.^1^^,^^5^ While the quality of the guiding literature is poor, it is clear that operator experience plays a crucial role in the of utility penile duplex ultrasound.^6^ However, its use is may be of value for surgical planning and for helping patients better understand the pathophysiology of their condition. Being able to identify the causative lesions and explain the physical deformity in real time to the patient while offering treatment options has been extremely beneficial in helping our patients understand their disease.

Current societal guidelines recommend the use of targeted intralesional therapies such as intralesional collagenase clostridium histolyticum that have shown impressive results in men with PD.^1^^,^^5^ Unfortunately, its use is difficult and there is no published data in men with normal physical exams and no palpable scars, requiring clinicians to seek alternative treatment options.

Given the referral structure of our clinic waiting times for a consultation can approach one year. Therefore, one may assume the symptoms were sufficiently bothersome to lead patients to present to their primary care practitioner and endure the lengthy waiting period before opting for conservative or minimally invasive therapy. In our cohort over 80% of ISS and over 90% of PS opted for continued observation after primary treatment. It appears education and providing an understanding of the source of the patients’ complaints, as well as its benign nature may play a crucial role in these patients. Observing the vascular flow and organic structural pathology may be reassuring to patients relieving them of emotional burden of suspecting a psychological source. Future studies should include more formal assessments of patient satisfaction.

The role of phosphodiesterase-5 inhibitors (PDE-5i) in PD remains controversial. While PDE-5i may work to inhibit fibrosis in animal models they have failed to show any appreciable effect on curvatures in men.^7^^,^^8^ Daily PDE-5i have been shown to improve International Index of Erectile Function scores and resolve septal scarring in 69% of ISS patients.^3^ Given the diffuse nature of PS it is unclear whether PDE-5i are having any identifiable impact on both the scarring and ED or ED alone, however with the high tolerability of this medication and the observed patient satisfaction, it has become a staple in our management.^3^

In our cohort of men with ISS we were able to identify potentially causative lesions on duplex ultrasound. This resulted in nearly 15% of men opting for intralesional therapy. Whether or not these ultrasonographic findings have any clinical implication is as of yet unknown. Intralesional verapamil may improve pain, curvature, and sexual function, though debate regarding its efficacy exist with minimal improvement in clinically relevant factors.^9^ Animal models have shown histological evidence of decreased collagen, decreased smooth muscle fibers, and increased penile pressures. It is feasible that these histologic changes result in meaningful improvements in men with ISS.^10^ Given the positive response to intralesional therapy it would be interesting to investigate the impact of other intralesional therapies such as collagenase inhibitors that have shown impressive results in men with palpable plaques.^11^

Surgical treatment through either reconstruction or inflatable penile prosthesis remains the gold standard in the treatment of PD. Given the diffuse nature of the scarring without an identified index lesion in most patients with PS, treatment of these patients can be challenging. While the data is limited, they appear to respond poorly to oral therapies and targeting with intralesional therapy can be difficult.^4^ These treatment difficulties, in combination with known concomitant ED, suggest early surgical intervention with penile prosthesis may be an appropriate option in severely symptomatic patients.

We acknowledge the significant limitations to our report, including the retrospective design and non-standardized measures of success. However, the large cohort of PS and ISS patients with normal physical exams provides some guidance to managing clinicians and raise further questions worthy of further study. We aim to complete a prospective study on the optimal management of these patients based on the results of this study to further investigate the clinical impact of ISS and PS on patient presentation and treatment outcomes.

## Conclusions

Patients with ISS and PS represent an under-recognized subset of PD that may require adjunctive diagnostic and modified treatment algorithms. In many patients a palpable scar may not be present, therefore identification and targeting of an intraseptal scar through duplex ultrasonography may prove useful in guiding therapy. Majority of men with ISS and PS were satisfied following conservative or minimally invasive therapies. Given the diffuse scarring and association with ED, clinicians may consider early penile prosthesis in patients with severely symptomatic punctate scarring, however in majority of men conservative therapy appears to be adequate.

## Statement of Authorship

Noah Stern: Conceptualization, Methodology, Investigation, Data curation, Writing – original, Writing – review, Visualization; Nahid Punjani: Conceptualization, Methodology, Formal analysis, Investigation, Data curation, Writing – review, Visualization; Gerald Brock: Conceptualization, Methodology, Resources, Data curation, Writing – review, Visualization, Supervision, Project administration.
